# Pyrrolidine dithiocarbamate (PDTC) inhibits the overexpression of MCP-1 and attenuates microglial activation in the hippocampus of a pilocarpine-induced status epilepticus rat model

**DOI:** 10.3892/etm.2013.1397

**Published:** 2013-11-08

**Authors:** RILANG LV, XIAOYUN XU, ZHENG LUO, NAN SHEN, FENG WANG, YONGBO ZHAO

**Affiliations:** 1 Department of Neurology, Shanghai East Hospital Affiliated to Tongji University School of Medicine, Shanghai 200210, P.R. China; 2 Department of Neurology, Shanghai Pudong New Area Zhoupu Hospital, Shanghai 201318, P.R. China; 3 Department of Neurology, Shanghai Jiaotong University Affiliated First People’s Hospital, Shanghai 200080, P.R. China

**Keywords:** epilepsy, pilocarpine-induced status epilepticus rat model, MCP-1 (CCL2), nuclear factor-κB, pyrrolidine dithiocarbamate, hippocampus

## Abstract

The aim of this study was to investigate the effects of pyrrolidine dithiocarbamate (PDTC) on MCP-1 expression and microglial activation in the hippocampus of a rat model of pilocarpine (PILO)-induced status epilepticus (SE). Moreover, seizure susceptibility, frequency and severity as well as brain damage were analyzed and changes in behavior were recorded. Chemokine MCP-1 expression and microglial activation were detected by immunohistochemistry (IHC). Fluoro-Jade C (FJC) and NeuN staining were used for the evaluation of tissue damage. Our results showed that although SE resulted in the upregulation of MCP-1 and microglial activation in the rat hippocampus 24 h after seizure onset, pretreatment with PDTC significantly inhibited the MCP-1 overexpression and attenuated the microglial activation. These effects were accompanied by neurodegenerative amelioration. To the best of our knowledge, these findings indicated for the first time that the activation of the nuclear factor-κB (NF-κB) pathway may contribute to MCP-1 upregulation and microglial activation in the context of epilepsy. PDTC was also shown to exert anticonvulsant activity and to have a neuroprotective effect on the hippocampal CA1 and CA3 regions, potentially through attenuating microglial activation.

## Introduction

As increasing evidence supports the hypothesis that brain inflammation is involved in the pathogenesis of epilepsy (1), the pivotal role of immune inflammatory reaction in epilepsy is increasingly being recognized (2). Pro-inflammatory chemokines, as chemotactic factors that control leukocyte migration under physiological and pathological conditions, are important components in neuro-inflammation. An increasing number of studies suggest that they play a critical role in epileptogenesis (3,4). For example, numerous chemokines and chemokine receptors, including CCL2/CCR2 (5), CCL3/CCL4 (6), CCL5 (7), CCR5 (8,9), CCR7/CCR8/CCR9/CCR10 (10), CXCL1 (11), CXCL12/CXCR4 (12) and CX3CL1/CX3CR1 (13,14) are implicated in epilepsy. Among them, CCL2 (also known as MCP-1) has been widely investigated in experimental rodents and clinical epileptic patients (5,15–18). It is considered that the inhibition of upregulated MCP-1 expression is likely to be beneficial in the treatment of refractory epilepsy (3,17,18).

Microglia, the resident macrophages in the brain parenchyma, play a key role in neuroinflammatory processes (19). Seizure induced by pilocarpine (PILO) results in rapid microglial activation in the rat hippocampus (20) and it is critical in the progression of neurodegeneration (21). However, it has been demonstrated that the amelioration of tissue damage and PILO-induced seizure severity may be achieved by attenuating microglial activation (22).

Pyrrolidine dithiocarbamate (PDTC), a selective nuclear factor-κB (NF-κB) inhibitor and antioxidant (23), exerts a significant anticonvulsant effect and has demonstrated a neuroprotective effect on the CA1 and CA3 regions of the hippocampus of a kainic acid (KA)-induced seizure rat model (24,25) as well as on the piriform cortex in the PILO status epilepticus (SE) model (26) by several mechanisms. However, whether PDTC protects against hippocampal damage by inhibiting MCP-1 upregulation and microglial activation in the PILO-induced SE model has not yet been investigated.

In the present study, to the best of our knowledge, we investigated the effects of PDTC on MCP-1 expression and microglial activation in the hippocampus of SE model rats for the first time. Moreover, seizure susceptibility, frequency and severity as well as brain damage were also analyzed.

## Materials and methods

### Ethical approval

All the experimental procedures were conducted according to the Guidance Suggestions for the Care and Use of Laboratory Animals formulated by the Ministry of Science and Technology of China (2006; Beijing, China). The study was approved by The Medicine and Life Science Ethics Committee of Tongji University (Shanghai, China).

### PILO-induced seizures

Approximately 40 adult male Sprague-Dawley rats (170–180 g) were purchased from Shang Hai Xipuer-BiKai experimental animals Co., Ltd. Prior to the experiment, the rats were housed for at least 1 week at a constant temperature of 22±1°C and relative humidity (60%) and had free access to standard food and water under a fixed 12-h light/dark cycle. Male Sprague-Dawley rats (230–250 g) were randomly allocated into three groups: i) the saline group (NS group), ii) the PILO-induced SE group (SE group), and iii) the SE with PDTC-pretreatment group (PDTC group). The rats of the SE group were treated with methylscopolamine (1 mg/kg, i.p; Sigma, St. Louis, MO, USA) 30 min prior to the pilocarpine hydrochloride i.p. injection (320 mg/kg; Sigma) (5). The rats of the PDTC group were pretreated with PDTC (100 mg/kg, i.p; Sigma) 24 h and 20 min prior to the administration of PILO. The rats of the NS group were injected with an equivalent volume of normal saline. The seizure activity was then scored according to the system developed by Racine (27). Only the animals that reached a seizure grade of ≥5 were selected for further analysis. All the animals successfully established were sacrificed for analysis 24 h after SE onset. Thirty-two rats were used in the analysis for seizure onset time, 27 rats were used in the analysis for the falling numbers, 15 rats were used in the analysis for the pattern of MCP-1 and tissue damage 24h after SE, and 5 rats were used in the analysis for the microglia activation in the three groups.

### Seizure observation

Following the administration of PILO, the behavioral changes of the rats were immediately observed using a video camera. The seizure onset time to grades 3 (SOT3) and 5 (SOT5) as well as the number of falls in the following 3 h after injecting PILO were recorded by 2 independent observers blinded to the sample identity. The seizure onset time to grade 3 and 5 were used to evaluate seizure susceptibility (28), while the number of falls was used to estimate seizure frequency and severity.

### Tissue processing

The rats were deeply anesthetized with 10% chloral hydrate and then transcardially perfused with saline followed by 4% paraformaldehyde. The brain was rapidly removed and post-fixed in the same fixative for 24 h followed by rinsing with phosphate-buffered saline (PBS) containing 30% sucrose at 4°C for ≥2 days (29). Tissue sections (50 μm thick) were cut using a cryostat.

### Double immunofluorescence staining

Fifteen rats were used for studying the pattern of MCP-1 24-h after SE. For double immunofluorescence analysis, after pretreatment with 0.01 M citrate buffer (pH 6.0) for 5 min at 95°C, the sections were incubated with a mixture of primary antibodies in PBS containing 0.3% Triton X-100 and 1% normal bovine serum for 1 h at room temperature (RT) followed by overnight incubation at 4°C. The primary antibodies were as follows: polyclonal goat antibody against MCP-1 (sc-1785, 1:100; Santa Cruz Biotechnology, Inc., Santa Cruz, CA, USA), mouse monoclonal antibody against NeuN (1:1,000; Chemi-Con, Rosemont, IL, USA), MCP-1 and polyclonal rabbit antibody against GFAP (1:500; DakoCytomation, Glostrup, Denmark). After washing three times with PBS for 15 min, the secondary antibodies donkey-anti-goat 488 (1:500; Invitrogen, Carlsbad, CA, USA), donkey-anti-rabbit-Cy3 (711-165-152, 1:500; Jackson ImmunoResearch, West Grove, PA, USA) and donkey-anti-mouse-Cy3 (715-165-150, 1:500; Jackson ImmunoResearch) were used to detect MCP-1, GFAP and NeuN. The tissues were then washed with PBS, mounted onto glass slides and coverslips were applied.

### Immunohistochemistry (IHC)

IHC was performed to investigate microglial activation. Briefly, all sections were initially incubated with 0.3% H_2_O_2_ for 15 min at RT. The sections were then incubated with rabbit anti-Iba1 polyclonal antibody (1:4,000; Wako Pure Chemical Industries, Ltd., Osaka, Japan) for 1 h at RT followed by overnight incubation at 4°C. Then, the biotinylated donkey anti-rabbit secondary antibody (711-065-152, 1:500; Jackson ImmunoResearch) was used for a 3-h incubation at RT. After washing three times for 5 min each, the sections were visualized with 3,3′-diaminobenzidine (DAB) in 0.1 M Tris buffer and mounted on gelatin-coated slides.

### Fluoro-Jade C (FJC) staining

Fifteen rats were used for studying tissue damage 24-h after SE. FJC staining was used to evaluate neuronal degeneration. The procedures were conducted as described by Wang *et al*(30).

### Cell counting

Cell counts were performed by two different investigators who were blind to the classification of tissues. Regarding MCP-1-positive cells, 3 sections throughout the bilateral hippocampus (at ~4.80–5.60 mm from the bregma) of each rat in the SE and PDTC groups were selected. Regarding FJC-positive cells, 5 or 6 sections throughout the bilateral hippocampus (at ~2.88–4.16 mm from the bregma) were used and the cells were counted with a ×10 objective magnification. All the cells in every region of each rat in the unilateral hippocampus were calculated using the following formula: Total number of positive cells counted in bilateral hippocampus/2 × corresponding cell numbers of sections. For the quantification of microglial cells, the methods described by Yeo *et al*(14) were used. All the immunoreactive cells were counted regardless of the intensity of labeling.

### Statistical analysis

Data are provided as the mean ± SEM. Statistical analysis was performed using SPSS software version 17.0 (SPSS, Inc., Chicago, IL, USA). All the data with the exception of the comparison of microglial activation were analyzed using an independent sample test, while one-way ANOVA was used to determine the statistical significance of microglial activation in the three groups. P<0.05 was considered to indicate a statistically significant difference.

## Results

### Pretreatment with PDTC enhances seizure susceptibility, but reduces the frequency and severity of PILO-induced seizures

**Figure 1:**
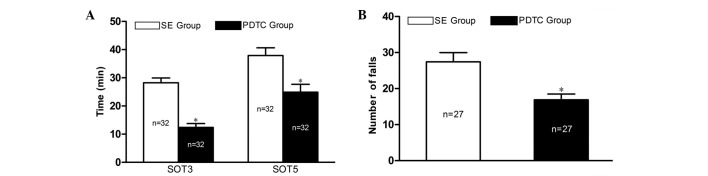
PDTC pretreatment reduces the seizure onset time in PILO-induced SE model rats, but alleviates the frequency and severity of PILO-induced seizures. (A) The mean seizure onset time to grades 3 (SOT3) and 5 (SOT5) as the latencies to seizure and (B) the number of falls as the frequency and severity of seizures of each group are shown. (A) SOT3 and SOT5 in the PDTC group were 12.4±1.4 and 24.9±2.8 min, respectively, compared with 28.2±1.7 and 37.9±2.8 min in the SE group. (B) The number of falls was 27.4±2.6 in the SE group and 16.9±1.6 in the PDTC group. ^*^P<0.01 vs. the SE group. PDTC, pyrrolidine dithiocarbamate; PILO, pilocarpine; SE, status epilepticus.

No seizures were observed in the rats of the NS group. The mean seizure onset times to grades 3 (SOT3) and 5 (SOT5) of the rats in the SE group were 28.2±1.7 and 37.9±2.8 min, respectively. The rats of the PDTC group exhibited significantly shorter SOT3 and SOT5 (12.4±1.4 and 24.9±2.8 min, respectively, P<0.01; Fig. 1A) compared with the rats of the SE group. However, PDTC attenuated the frequency and severity of the PILO-induced seizures, which was reflected by a significantly reduced number of falls in the PDTC group (16.9±1.6 vs. 27.4±2.6, P<0.01) during the 3 h following the injection of PILO (Fig. 1B).

### PDTC exerts a neuroprotective effect on the hippocampal CA1 and CA3 regions of PILO-induced SE model rats

**Figure 2:**
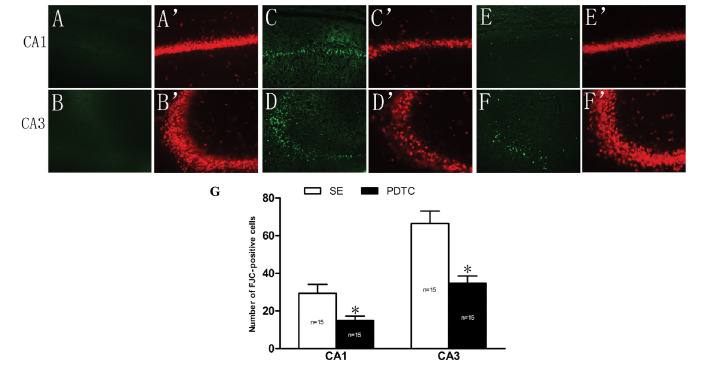
Effect of PDTC pretreatment on PILO-induced neuronal damage in the hippocampal CA1 and CA3 regions. FJC staining in (A and B) the NS group, (C and D) the SE group and (E and F) the PDTC group; NeuN staining in (A′ and B′) the NS group, (C′ and D′) the SE group and (E′ and F′) the PDTC group, SE resulted in significant neuronal damage in the hippocampal CA1 and CA3 regions compared with that in the NS group (A, B, A′ and B′), since a large number of FJC-positive cells were detected by FJC staining and many neurons were shown to be missing by NeuN staining (C, D, C′ and D′). The number of FJC-positive cells was 29.4±4.7 and 66.5±6.6 in the CA1 and CA3 regions of the rats in the SE group, respectively. By contrast, the numbers of FJC-positive cells in the CA1 (15.0±2.3) and CA3 (34.6±3.9) regions were significantly reduced in the PDTC group (P<0.01) (E and F), which was also confirmed by NeuN staining (E′ and F′). (G) Quantitative analysis of the number of FJC-positive cells in the CA1 and CA3 regions of the rats in the SE and PDTC groups (mean ± SEM). ^*^P<0.01 vs. the SE group. PDTC, pyrrolidine dithiocarbamate; PILO, pilocarpine; SE, status epilepticus; NS, saline-treated; FJC, Fluoro-Jade C. (A–F′) Magnification, ×200.

In the NS group, no FJC-positive cells were detected in the CA1 and CA3 regions of the rat hippocampus (Fig. 2A and B). In addition, NeuN staining indicated that the pyramidal cells of the hippocampal CA1 and CA3 regions were structurally intact with closely aligned cells and clear nuclei (Fig. 2A′ and B′). However, 24 h after SE, the hippocampal structures were markedly damaged since numerous FJC-positive cells were detected and many neurons were shown to be missing by NeuN staining (Fig. 2C, C′, D and D′). The numbers of FJC-positive cells in the CA1 and CA3 regions were significantly reduced in the PDTC group compared with those in the SE group (15.0±2.3 vs. 29.4±4.7 and 34.6±3.9 vs. 66.5±6.6, respectively; P<0.01; Fig. 2E, F and G). Opposite results were obtained following NeuN staining (Fig. 2E′ and F′). In the PDTC group, the NeuN staining indicated that the structure of the pyramidal cells of the hippocampal CA1 and CA3 were damaged and small cells were missing.

### Increased chemokine MCP-1 expression in the rat hippocampus following PILO-induced SE is inhibited by PDTC pretreatment

**Figure 3:**
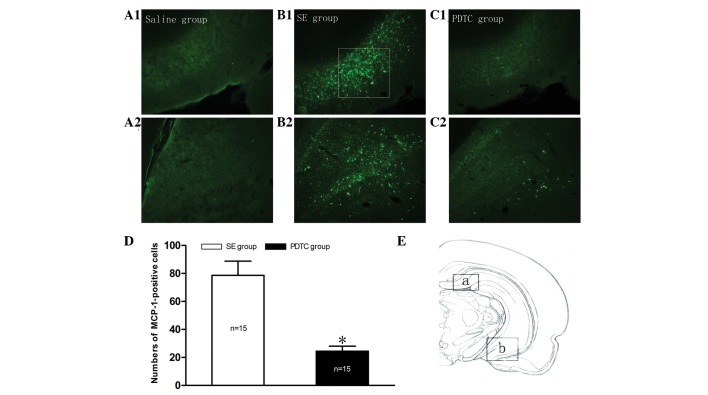
MCP-1 expression in the hippocampal tissues of the rats in each group. No hippocampal MCP-1 expression was detected in the rats in the NS group (A1 and A2). However, the numbers of MCP-1 immunoreactive cells were significantly increased in these regions of the rats in the SE group compared with those in the NS group (B1 and B2). The numbers of MCP-1-positive cells were significantly reduced in the PDTC group (C1 and C2) compared with those in the SE group. (D) Quantitative analysis indicated that the relative numbers of hippocampal MCP-1-positive cells were 78.6±10.2 in the SE group and 24.5±3.6 in the PDTC group (mean ± SEM). ^*^P<0.01 indicates a significant difference from the SE group. (E) The two regions (a and b) in the hippocampus that are shown in A1–C1 and A2–C2, respectively. NS, saline-treated; SE, status epilepticus; PDTC, pyrrolidine dithiocarbamate. (A1–C2) Magnification, ×200.

**Figure 4:**
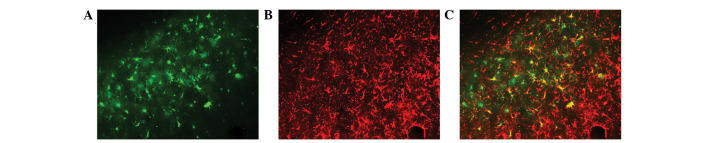
Double immunofluorescence staining for MCP-1 and GFAP in the hippocampus of SE model rats. MCP-1 immunoreactive cells were co-localized with GFAP (C), suggesting that SE resulted in the upregulation of MCP-1, which was mainly expressed by astrocytes. (A) MCP-1-positive cells; (B) GFAP-positive cells; (C) co-localization of MCP-1 and GFAP. Magnification, ×400.

No MCP-1 immunolabeled cells were observed in the hippocampus of the rats in the NS group (Fig. 3A1 and A2). However, as shown in Fig. 3B1 and B2, MCP-1 was steadily expressed in the two regions of the hippocampus (boxed areas a and b in Fig. 3E) 24 h following PILO-induced SE. Therefore, these areas were selected for further study and quantitative analysis. As shown in Figs. 3 and 4, SE resulted in MCP-1 overexpression and almost all the positive cells were co-localized with GFAP (Fig. 4C). However, the overexpression of MCP-1 was markedly suppressed by PDTC pretreatment (Fig. 3C1 and C2). Quantitative analysis indicated that the relative total number of MCP-1 immunopositive cells in the PDTC group was significantly reduced compared with that in the SE group (24.5±3.6 vs. 78.6±10.2, respectively; P<0.01; Fig. 3D).

### PDTC inhibits microglial activation in the CA1 and CA3 regions of the hippocampus in PILO-induced SE rats

**Figure 5:**
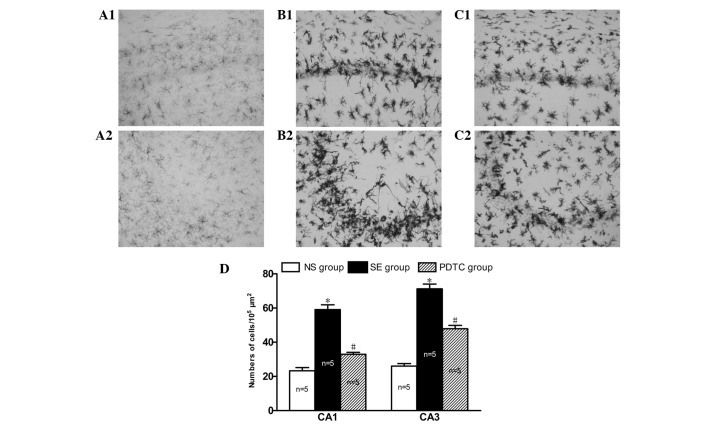
State of microglial activation in the three groups of rats. (A1–C1) Hippocampal CA1 and (A2–C2) hippocampal CA2 regions. Microglia showed resting states with thin cell bodies and slender processes in the NS group (A1 and A2). In the SE group, the numbers of activated microglial were evidently increased in the CA1 and CA3 regions of the hippocampus compared with those in the NS group (B1 and B2). However, in the PDTC group, the numbers of activated microglia in the CA1 and CA3 regions were significantly reduced (C1 and C2). (D) Quantitative analysis of the number of activated microglia cells in the CA1 and CA3 regions of the rats in the three groups (mean ± SEM).^*^P<0.01 vs. the NS group; ^#^P<0.01 vs. the SE group. (A–C) Magnification, ×400.

Microglia cells were equally distributed and exhibited resting states with thin cell bodies and slender processes in the CA1 and CA3 regions of the hippocampus in the rats in the NS group (Fig. 5A1 and A2). However, 24 h after SE, the microglia were activated, demonstrating a hyper-ramified and amoeboid or phagocytic appearance (Fig. 5B1 and B2). Notably, the numbers of activated microglia migrating to the death-susceptible pyramidal neuron cell layers CA1 (59.1±6.2 vs. 23.3±4.2, P<0.01) and CA3 (71.3±6.3 vs. 26.1±3.2, P<0.01) were significantly increased in the SE group compared with the NS group (Fig. 5D). However, the accumulation of activated microglia at these sites was significantly reduced in the PDTC group compared with the SE group (33.0±2.6 vs. 59.1±6.2, respectively, P<0.01 and 47.9±4.3 vs. 71.3±6.3, respectively, P<0.01; Fig. 5C1, C2 and D).

## Discussion

The present study demonstrated that: i) pretreatment with PDTC enhances seizure susceptibility and exerts an anticonvulsant action; ii) PDTC has a neuroprotective effect on the hippocampal CA1 and CA3 regions of rats with PILO-induced SE; and iii) PDTC inhibits MCP-1 overexpression and attenuates microglial activation in the hippocampus of the PILO-induced SE model rats.

PDTC, a selective NF-κB inhibitor and antioxidant (23), has been previously demonstrated to exert anticonvulsant activity and to have a neuroprotective effect through stimulating the adenosine A1 receptor (25) and antagonizing the activated NOS II-peroxynitrite signaling cascade (24) in a KA-induced seizure model. Our data showed that PDTC enhanced seizure susceptibility, as indicated by the shorter SOT3 and SOT5, but reduced the frequency and severity of the PILO-induced seizures and protected the CA1 and CA3 regions of the hippocampus against neurodegeneration. In the present study, the observation that PDTC enhanced seizure susceptibility was similar to the findings of Lubin *et al*(31) but not in agreement with those by Yu *et al*(28). Lubin *et al*(31) suggested that PDTC is related to the reduced expression of brain-derived neurotrophic factor (BDNF) gene following inhibition of the NF-κB pathway, while Yu *et al*(28) observed that PDTC significantly extended the seizure onset time. The contrasting data from Lubin *et al*(31) and Yu *et al*(28) may be related to the different drugs tested (DDTC and SN50 vs. PDTC) as the same SE model (the KA model) was used in both. The contradictory results between the present study and the study by Yu *et al*(28) may be related to the different SE models used (the PILO-induced seizure model vs. the KA model), since the same drug (PDTC) was used. It is not possible to determine the idiographic mechanisms of these contradictory findings in this study and further investigation is required. Furthermore, the current study demonstrated that PDTC reduced the frequency and severity of PILO-induced seizures. The following three aspects should be considered: i) PDTC has a protective effect on the hippocampus. Shin *et al*(25) demonstrated that PDTC reduced the number of animals developing generalized SE through stimulating the adenosine A1 receptor and then blocking seizure-induced oxidative stress and subsequent neuronal loss (25). ii) PDTC alleviates brain inflammation. Liu *et al*(32) reported that PDTC exerted an antiepileptic effect by inhibiting the NF-κB pathway and thereby reducing the expression levels of NF-κB/P65, tumor necrosis factor-α (TNF-α), interleukin (IL)-β and IL-10 in the hippocampus. iii) PDTC attenuates microglial activation. Abraham *et al*(22) showed that PDTC alleviates seizures by attenuating microglial activation.

MCP-1 may act as a modulator of neuronal activity and neuroendocrine functions in the brains of normal rats (33). However, it has been reported that MCP-1 protein and mRNA levels as well as those of its receptor, CCR2, are significantly increased in the brain tissues of epileptic patients or experimental rodents (5,15–18). The cell types expressing MCP-1 were mainly neurons and astrocytes 24 h after SE (17), and mainly microglia 2 days post SE (15). In the present study, the numbers of the MCP-1 immunopositive cells were significantly increased in the rat hippocampus and were mainly expressed by astrocytes 24 h after PILO-induced SE. The difference in the cell types expressing MCP-1 may be associated with the different time points after SE. However, the mechanism underlying the differential expression of MCP-1 in various types of cells at different time points after SE remains be elucidated, as the available literature was not investigated. However, the upregulation of MCP-1 was attenuated in rats pretreated with PDTC. This may be explained by the selective NF-κB inhibition mechanism of PDTC. It has been shown that inflammation induces the expression of MCP-1 through the NF-κB signaling pathway under several pathological conditions, such as progressive proteinuric nephropathy (34), cardiac ischemia-reperfusion injury (35) and high glucose stimulation (36). Meanwhile, the NF-κB signaling pathway has been shown to be involved in MCP-1 gene regulation (37). Therefore, the results obtained in the present study indicate that the inhibition of MCP-1 overexpression by PDTC in the context of epilepsy is likely to be associated with the inhibition of NF-κB activation. Even though PDTC exerts a dual mechanism as an NF-κB inhibitor and antioxidant, the main mechanism by which PDTC acts in SE is likely to be the inhibition of NF-κB since the pharmacological effect of PDTC has been shown to be similar to that of double-stranded κB decoy DNA which is a selective NF-κB inhibitor, while its antioxidant properties are negligible (24). Yu *et al*(28) confirmed that PDTC pretreatment significantly decreased NF-κB activation 24 h after KA-induced SE.

Furthermore, to the best of our knowledge, our data showed for the first time that PDTC attenuated microglial activation and ameliorated neurodegenerative changes in the hippocampus in SE model rats. Microglia cells became hyper-ramified from their resting states after SE, indicating the expression of inflammatory factors and the induction of cytotoxic activity (38). In addition, activated microglia synthesized and released IL-1β (39), osteopontin (OPN) (40), telomerase (41), IL-6 and TNF-α (42), which contribute to the occurrence of brain injury. This may explain the increased number of activated microglia that migrated to the death-susceptible pyramidal neuron cell layers CA1 and CA3 24 h after SE. Regarding the attenuation of microglial activation and ameliorative neurodegeneration, three aspects should be considered. Firstly, these effects may be related to the inhibition of MCP-1 since activated microglia migrate to areas of injury guided by chemokines in the inflammatory process (11) and MCP-1 was critical for the microglia migration and subsequent neurodegeneration (17). Secondly, the dual mechanism of action of PDTC should also be considered. PDTC was previously demonstrated to affect microglia-mediated neuroinflammation through inhibiting ROS and NF-κB pathways (43). Thirdly, the neuroprotective effect of PDTC may be related to the inhibition of microglial function and the reduction of cytokine releasing or cytotoxic activity.

However, the present study has several limitations. Firstly, the activation of NF-κB in each group was not investigated, since this has been previously examined in other studies (24,28,31). Secondly, the effects of PDTC on MCP-1 mRNA expression in the context of epilepsy was not investigated, which will be examined in future studies by our group.

In conclusion, the present study demonstrated that the protective effects of PDTC on hippocampal damage may be associated with inhibited microglial activation in the PILO-induced SE rat model. To the best of our knowledge, these results indicate for the first time that the activation of the NF-κB pathway contributes to MCP-1 upregulation and microglial activation under the context of epilepsy.
